# Systemic acidemia impairs cardiac function in critically Ill patients

**DOI:** 10.1016/j.eclinm.2021.100956

**Published:** 2021-06-29

**Authors:** S Rodríguez-Villar, JA Kraut, J Arévalo-Serrano, SG Sakka, C Harris, I Awad, M Toolan, S Vanapalli, A Collins, A Spataru, P Eiben, V Recea, C Brathwaite-Shirley, L Thompson, B Gurung, R Reece-Anthony, The Acid-Base Working Group.

**Affiliations:** a Critical Care Department, King´s College Hospital NHS Trust Foundation. London, United Kingdom; b Division of Nephrology and Department of Medicine Veterans Administration Greater Los Angeles Healthcare System and UCLA School Of Medicine, California, United States; c Internal Medicine Department, Hospital Príncipe de Asturias, Alcalá de Henares, Madrid, Spain; d Critical Care Department. Gemeinschaftsklinikum Mittelrhein gGmbH, Kemperhof und Ev, Stift St. Martin. Academic Teaching Hospital of the Johannes Gutenberg University Mainz. Germany; e Critical Care Department. Queen Elizabeth Hospital, Lewisham and Greenwich NHS Trust, United Kingdom; f Critical Care Department. Princess Royal University Hospital, King´s College Hospital NHS Trust Foundation, London, United Kingdom; g Critical Care Department. Lewisham University Hospital, Lewisham and Greenwich NHS Trust, United Kingdom

**Keywords:** Acidosis, Acidemia, pH, ABG-a, Cardiac contractility, Cardiac function, CI, CO, SVI, CFI, GEF, dPmx, CPI, PiCCO, Transpulmonary thermodilution, Critical care, Point-of-Care arterial blood gas

## Abstract

**Background:**

Acidemia, is associated with reduced cardiac function in animals, but no studies showing an effect of acidemia on cardiac function in humans are reported. In the present study, we examined the effect of acidemia on cardiac function assessed with transpulmonary thermodilution technique with integrated pulse contour analysis (Pulse Contour Cardiac Output, PiCCO™) in a large cohort of critically ill patients.

**Methods:**

This was a prospective multicenter observational cross-sectional study of 297 patients from 6 intensive care units in London, England selected from all patients admitted consecutively between May 2018 and March 2019. Measurements of lowest plasma pH and concurrent assessment of cardiac function were obtained.

**Findings:**

There was a significant difference between two pH categories (pH ≤ 7.28 vs. pH > 7.28) for the following variables of cardiac function: SVI (difference in means 32.7; 95% CI: 21 to 45 mL/m2; *p* < 0.001); GEF (18; 95% CI: 11 to 26%; *p* < 0.001), dPmax (-331; 95% CI: -510 to -153 mmHg/s; *p* = 0.001), CFI (0.7; 95% CI: 0.2 to 1.3 1/min; *p* = 0.01) and CPI (0.09; 95% CI: 0.03 to 0.15 W/m2; *p* < 0.001). However, there was no significant difference in CI (0.13; 95% CI: -0.20 to 0.47 L/min/m2; *p* = 0.12) between the pH categories. Also, a significant relationship was found between the quantitative pH and the following variables: SVI (132; 95% CI: 77 to 188 mL/m2; *p* < 0.001), GEF (74.7; 95% CI: 37.1 to 112.4%; *p* < 0.001), dPmax (-1587; 95% CI: -2361 to -815 mmHg/s; *p* < 0.001), CFI (3.5; 95% CI: 0.9 to 6.1 /min; *p* = 0.009), CPI (0.62; 95% CI: 0.36 to 0.88 W/m2; *p* < 0.001) and CI (regression coefficient 1.96; 95% CI:0.45 to 3.47 L/min/m2; *p* = 0.01).

**Interpretation:**

Acidemia is associated with impaired cardiac function in seriously ill patients hospitalized in the intensive care unit supporting the potential value of early diagnosis and improvement of arterial pH in these patients.

**Funding:**

The study was partially supported by unrestricted funds from the UCLA School of Medicine.

**Research in context**

**Evidence before this study**

Review of the literature from 1968 to the present revealed studies in animals using sensitive methods to assess cardiac function showed acidemia was associated with impaired cardiac contractility and cardiac function. However, there were no studies in humans using similar sensitive methods to confirm these findings.

**Added value of this study**

This is the first study performed in a large cohort of seriously ill patients which examined the impact of acidemia on cardiac function using the PiCCO system, a sophisticated method to assess cardiac function. The study reveals systemic acidemia was associated with impaired cardiac function.

**Implications of all the available evidence**

The results of this study indicate that systemic acidemia in humans is associated with impaired cardiac function and suggest that amelioration of the acidemia could improve cardiac function and theoretically improve clinical outcome.

Alt-text: Unlabelled box

## Introduction

Lactic acidosis with acidemia is common in seriously ill patients hospitalized in the intensive care unit (ICU), particularly those with underlying sepsis. Epidemiological studies have shown the incidence of acidemia can vary from 14% to as high as 42% [1]. The acidemia is often associated with a poor prognosis: mortality can be as high as 57% when the acidemia is severe. The increased mortality has been ascribed, in part, to impairment of cardiac function by the systemic acidemia causing reduced tissues perfusion of vital tissues [2,3]. This assumption is primarily based on extrapolation from results of studies obtained in dogs in which reduced cardiac contractility was observed when severe acidemia was produced by infusion of lactic acid [4,5]. However, no studies have examined the effect of systemic acidemia on cardiac function in a large cohort of seriously ill humans [6].

Therefore, given the potential impact of acid-base disorders on clinical outcome in patients hospitalized in the ICU, there remains an unmet need for investigations focusing on the impact of systemic acidity on cardiac function in these patients.

In the present study, we examined the relationship between systemic acidemia and several measures of cardiac function obtained using a sensitive and specific monitoring device, the transpulmonary thermodilution technique with integrated pulse contour analysis (PiCCO™, Pulsion, Maquet Getinge Group) in 297 seriously ill patients admitted to six ICUs from hospitals in London, England.

## Methods

### Study design and patients

This was a prospective multicenter observational cross-sectional study. This study is reported following the Strengthening The Reporting of Observational Studies in Epidemiology (STROBE) statement. Local investigators screened eligible patients from six ICU's of hospitals the King´s College NHS Foundation Trust and Lewisham and Greenwich NHS Trust, London, England. For accuracy, reproducibility, and consistency, we standardized the inclusion and exclusion criteria used for patient recruitment (Table 1; Appendix). All patients admitted consecutively between May 2018 and March 2019 who were 18 or older, were in sinus rhythm, required invasive hemodynamic monitoring and were under controlled mechanical ventilation via an endotracheal tube (minimum tidal volume 6–8 mL/Kg) were included in the study. Patients with atrial or ventricular arrythmias, valvular abnormalities, intracardiac shunts, who were being treated with extracorporeal membrane oxygenation, or did not require mechanical ventilation, were excluded.

**Table 1:** Characteristics, chronic disease and admitting diagnosis of pH groups ≤ 7.28 and > 7.28.

	pH ≤ 7.28 n (%) or Md (IQR)*	pH > 7.28 n (%) or Md (IQR)*	*P*-value
Patients	153 (51.5)	144 (48.5)	
Sex Male	87 (56.9)	63 (43.8)	0.02
Age (years)	60 (50–71)	58 (49–70)	0.18
APACHE II score	23 (17–28)	19 (14–24)	< 0.001
Constant urine output > 0.5 mL/kg/h	24 (15.9)	64 (45.1)	< 0.001
Duration of ICU treatment (days)	10 (4 - 19)	11 (4–21)	> 0.20
Death at day 28	91 (60,3)	36 (25.4)	< 0.001
**Chronic diseases**			
Ischaemic heart disease	21 (13.7)	34 (23.6)	0.02
Congestive heart failure	10 (6.5)	9 (6.3)	> 0.20
Atrial fibrillation/flutter	4 (2.6)	11 (7.6)	0.06
Arterial hypertension	39 (25.5)	42 (29.2)	> 0.20
Pulmonary hypertension	3 (2.0)	1 (0.7)	> 0.20
Pulmonary embolus/Deep vein thrombosis	6 (3.9)	2 (1.4)	> 0.20
COPD/Asthma	36 (23.5)	27 (18.7)	> 0.20
Pulmonary fibrosis	1 (0.7)	1 (0.7)	> 0.20
Diabetes mellitus	30 (19.6)	39 (27.1)	0.13
Chronic kidney disease	9 (5.9)	18 (12.5)	0.091
Liver disease	27 (17.6)	24 (16.7)	> 0.20
Morbid obesity	7 (4.6)	4 (2.8)	> 0.20
Non-Haematological malignancies	11 (7.2)	7 (4.9)	> 0.20
**Admission diagnosis**			
Cardiogenic shock/Cardiac arrest N (%)	45 (29.4)	34 (23.6)	> 0.20
Cardiac surgery	1 (0.7)	9 (6.3)	0.01
Septic shock (medical)	30 (19.6)	13 (9.0)	0.01
Septic shock (neutropenic)	4 (2.6)	1 (0.7)	> 0.20
Septic shock (surgical)	26 (17.0)	17 (11.8)	> 0.20
Post-operative management	25 (16.3)	31 (21.5)	> 0.20
Haemorrhagic shock			> 0.20
Post-surgical	1 (0.7)	2 (1.4)	
Gastrointestinal/Other medical	7 (4.6)	4 (2.8)	
Major trauma	9 (5.9)	8 (5.6)	
Acute respiratory failure			> 0.20
Pneumonia	14 (9.2)	7 (4.9)	
COPD/Asthma	7 (4.6)	6 (4.2)	
Other	12 (7.8)	9 (6.3)	
Acute renal failure	30 (19.6)	29 (20.1)	> 0.20
Trauma/Neurosurgical	7 (4.6)	23 (16.0)	0.001
Decompensated liver disease/Acute liver failure	27 (17.6)	21 (14.6)	> 0.20
Other admitting diagnosis	2 (1.)	6 (4.2)	0.16

*Values are frequency count (N) and percent (%) for categorical variables or median (Md) and Interquartile range (IQR) for quantitative variables.

**Fig. 1:**
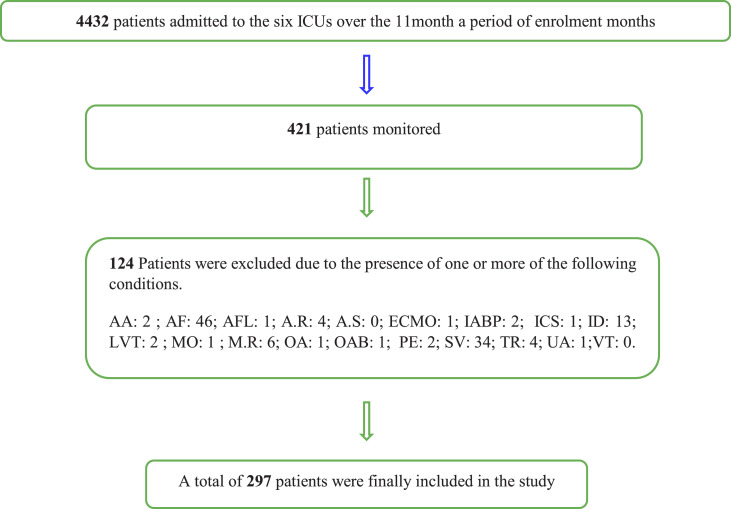
**Flow diagram of cohort selection.** AA (Severe Aortic Aneurism); AF (Atrial Fibrillation); AFL (Atrial Flutter); A.R (Severe Aortic Regurgitation); A.S (Severe Aortic stenosis); ECMO (Extracorporeal Membrane Oxygenation); IABP (Intra-aortic balloon pump); ICS (Intracardiac Shunt);ID (incomplete Data); LVT (Low Tidal Volume < 6–8 ml/kg); MO (Morbidity Obesity); M.R (Severe Mitral regurgitation); OA (Other Arrhythmias); OAB (Open Abdomen); PE (Pulmonary Embolus); SV (self-ventilation); TR (Severe Tricuspid Regurgitation); UA (Under Age, < 18 years of age).VT (Ventricular tachycardia).

Four hundred twenty-one patients being monitored with the PICCO™ system during the enrolment period were considered for inclusion. Two hundred ninety-seven patients who met the inclusion and exclusion criteria were included in the study (Fig. 1 and Table 1; Appendix). In addition, the following data were recorded (Table 1). The procedures (such as devices used, and measurements undertaken) were the same across all ICU sites.

### Ethical approvals

This was a prospective multicenter observational cross-sectional study. Local investigators screened eligible patients from six ICU's of hospitals the King´s College NHS Foundation Trust and Lewisham and Greenwich NHS Trust. In London, England. The study conformed to the Declaration of Helsinki and to local applicable regulatory provisions. The study was approved by both the local Research and Development department and the Regional Ethics committee (R&D number-KCH N/A; REC number 18/EM/0138 and IRAS project ID 227,870). The study is registered on the ClinicalTrials.gov public website with identifier NCT04231045.

### Consent

A proportionate approach to seeking consent was granted by the Health Research Authority. For our research participants there were no difference from the standard care they may expect to receive if they were not taking part.

### Procedures

Patients were monitored by the transpulmonary thermodilution technique with integrated pulse contour analysis Pulse Contour Cardiac Output (PiCCO™; Pulsion, Maquet Getinge Group, Feldkirchen, Germany). The decision to initiate monitoring using PiCCO™ was at the discretion of the treating physician. A central venous catheter was placed via the internal jugular or subclavian into the superior vena cava, and an arterial catheter was inserted into the femoral (PiCCO™ catheter 5F length 20 cm, PV2015L20-A) or axillary artery (PiCCO™ catheter 4F length 16 cm, PV2014L16-A).

After setting up the system, transpulmonary thermodilution (TPTD) and arterial pulse contour techniques (PiCCO™; Pulsion, Maquet Getinge Group, Feldkirchen, Germany) were used for hemodynamic monitoring as per departmental guidelines.

Pulse contour (PC) analysis provides continuous information while TPTD provides static measurements used to calibrate the continuous PC parameters. PiCCO™ calibration pre- and post-cold fluid administration was performed as per institutional practice by taking the average of 3 consecutive thermodilution cardiac output (CO) measurements. Each measurement was obtained by injection of 20 mL of cold 0.9% NaCl with a temperature of < 8 °C. Injections were made via the distal port of the central venous catheter and transpulmonary thermodilution curve was recorded by the thermistor embedded in the femoral or axillary arterial catheter. If the difference between the three values was > 10%, two additional measurements were performed subsequently. By means of transpulmonary thermodilution, the PiCCO™ system calculates CO using the modified Stewart-Hamilton equation, the mean transit time (MTt), and the exponential downslope time (DSt) of the curve. The product of CO and MTt gives the intrathoracic thermal volume (ITTV). The product of CO times the DSt gives the pulmonary thermal volume (PTV). The difference between ITTV and PTV is called global end-diastolic volume (GEDV), or GEDVI if indexed for the body surface area. Multiplying GEDV by 1.25 approximates the intrathoracic blood volume (ITBV). Stroke volume (SV) is calculated by dividing CO by heart rate. A 'global' ejection fraction (GEF) can be obtained by dividing SV by a quarter of GEDV. Similarly, dividing CO by the preload parameter GEDV gives an indicator of cardiac systolic function, the cardiac function index (CFI). Both, GEF and CFI are two transpulmonary thermodilution-derived indices of cardiac systolic function which is automatically calculated by the monitor. Both indices are therefore global ejection phase indices since they are the ratio of cardiac output or stroke volume to the global end-diastolic volume of the heart and are physiologically close to LV fractional area of change (LVFAC), which is the ratio of LV stroke area to LV end-diastolic area (an index of LV preload). CPI represents the power of left ventricular cardiac output in Watt/m². It is the product of pressure (MAP) and flow (CO) [7].

Steepness of the upward part of the arterial pressure curve (dPmax) is regarded as a marker of left ventricular contractility. The pressure changes during the systolic phase and a measure of the pressure increase over time (analyzed in speed) is calculated.

Extravascular lung water (EVLW) is equivalent to the difference between ITTV and ITBV. The ratio of EVLW and pulmonary blood volume is used as an index of pulmonary vascular permeability (PVPI) (Table 2; Appendix).

**Table 2:** Constants, blood gasses and laboratory values of pH categories ≤ 7.28 and > 7.28.

	pH ≤ 7.28 Md (IQR)*	pH > 7.28 Md (IQR)*	*P*- value
Constants			
Body temperature ( °C)	36.5 (35.5–37.4)	36.8 (36.4–37.6)	0.001
Heart rate (bpm)	104 (89–115)	88 (74–103)	< 0.001
Systolic blood pressure (mmHg)	108 (98–120)	115 (104–128)	0.001
Diastolic blood pressure (mmHg)	53 (49–59)	57 (52–66)	< 0.001
Mean blood pressure (mmHg)	68 (64–75)	76 (69–86)	< 0.001
central venous pressure (mmhg)	14 (11–18)	13 (9–17)	0.03
gases analysis			
Inspiratory oxygen fraction (%)	45 (35–65)	35 (30–45)	< 0.001
Oxygen saturation (%)	96 (93–99)	97 (95–99)	0.1
End-tidal CO_2_ tension (kPa)	4,3 (3,5 −5,4)	4,4 (3,9 - 5,1)	> 0.20
Arterial CO_2_ tension (kPa)	6,0 (5,2–7,2)	5,6 (5,1–6,1)	< 0.001
Arterial O_2_ tension (kPa)	11,7 (9,9–14,9)	11,5 (10,2–14,1)	> 0.20
Bicarbonate (mmol/L)	17,5 (13,6–20,6)	22,5 (20,5–25,0)	< 0.001
Bases excess (mmol/L)	−8,5 (−13,9 - −5,2)	−2,2 (−4,6–0,5)	< 0.001
Arterial oxygen saturation (%)	95,0 (92,1–97,0)	96,3 (94,6–97,8)	< 0.001
Laboratory values			
Sodium (mmol/L)	138 (135–142)	138 (136–142)	> 0.200
Chloride (mmol/L)	108 (106–112)	108 (105–111)	0.09
Potassium (mmol/L)	4.6 (4.2–5.1)	4,4 (4.2–4.8)	0.03
Ionized calcium (mmol/L)	1.17 (1.10–1.23)	1.18 (1.13–1.26)	0.03
Blood glucose (mmol/L)	7.6 (6.0–10.0)	8.3 (6.8–9.9)	0.05
lactate (mmol/l)	4.5 (2.1–10.5)	1.6 (1.0–2.7)	< 0.001
hemoglobin (g/l)	95 (81–115)	95 (84–107)	> 0.20
Urea (mmol/L)	8.9 (6.1–13.3)	8.9 (5.8–13.6)	> 0.20
Creatinine (mmol/L)	122 (90–182)	113 (74 to 164)	0.07
Magnesium (mmol/L)	0.99 (0.84–1.18)	0.95 (0.86–1.13)	> 0.200
Plasma total calcium (mmol/L)	2.03 (1.91–2.18)	2.06 (1.96–2.22)	0.05
Plasma total calcium corrected (mmol/L)	2.31 (2.17–2.47)	2.31 (2.20–2.49)	> 0.20
Phosphate (mmol/L)	1.49 (1.04–1.90)	1.16 (0.91–1.47)	< 0.001
Total protein (g/L)	48 (41–55)	50 (43–57)	0.03
Albumin (g/L)	26 (22 - 30)	27 (24 - 31)	> 0.20

*Values are median (Md) and Interquartile range (IQR).

All precautions were taken to avoid variability during measurements of cardiac output. The doses of vasopressors and inotropic agents were unchanged during the set of 3 measurements. Both pressure-preset, time-cycled ventilation (pressure control ventilation), and flow-controlled, volume-cycled ventilation with decelerating flow profile were used. Choice of ventilation mode was left at the discretion of the treating physician. Plateau pressure therefore was similar but not equal to peak airway pressure depending on whether inspiratory flow reached the zero line. Drop to zero inspiratory flow was not an a-priori requirement in this study. Lung compliance was not further characterized as static or dynamic. The only limiting ventilation parameter in this study was tidal volume, only patients with a minimum of 6–8 mL/kg according to patient's ideal body weight were recruited.

Measures of cardiac contractility, preload, and afterload derived from use of PiCCO™ and selected biochemical and physiological parameters were recorded for each patient when the lowest plasma pH in each patient was recorded.

### Outcomes

The primary objective of the study was to examine the relationship between systemic blood pH and cardiac function: particularly to identify and quantify any depressive impact of acidemia on measures of cardiac function. Cardiac function measurements included: stroke volume index, global ejection fraction, dPmax as marker of left ventricular contractility, cardiac function index, cardiac power index, and cardiac index. Cardiac function measurements were obtained at the lowest arterial pH obtained during each patient's ICU stay.

Decisions about evaluation and treatment were primarily made by the patient's clinician with the goal of improving the patient's clinical state. Therefore, establishment of monitoring by PiCCO was often delayed to allow rapid initiation of appropriate therapy. Thus, periods of severe acidemia could not always be examined. Based on the data available, we divided the patients into two groups with similar sample size (pH ≤ 7.28, *N* = 153 vs pH > 7.28, *N* = 144).

### Statistical analysis

The required sample size necessary for the study was based on results of the cardiac function index. Assuming a type I error rate of 5%, a type II error rate of 20% (beta risk, power of 80%) and a common standard deviation of 2.2 L/min for cardiac function index [8], we postulated we would need a minimum of 274 patients to detect a difference of 0.75 L/min in mean values between plasma pH ≤ 7.28 and > 7.28 group with 80% power. Assuming a loss to follow up rate of 5% during the study, we recruited 288 patients.

Summary statistics were reported, median (IQR) for numerical variables because they didn't need the normality, and frequency count (%) for categorical variables. Pearson Chi-square test or Fisher's exact test, as appropriate, were used to test for a significant association between categorical variables. Student's t-test or Mann-Whitney U test, as appropriate, were used to examine for a significant difference of quantitative variables between two groups.

To further examine the relationship between cardiac function and arterial pH, estimative linear regression analysis was performed using measures of cardiac function as the dependent variable and arterial pH, dichotomized or quantitative, as the independent variable. A scatter plot with fitted regression line was generated to visualize the relationship between these variables.

Model assumptions (linearity, independence, normality and equality of variances) were evaluated by examining the residuals.

Selection and confusion bias were controlled using Propensity Score Matching (PSM) were calculated from logistic regression with the pH group as the dependent variable and the following as independent variables: sex, APACHE II score, body temperature, systolic blood pressure, noradrenaline, pCO_2_, arterial [HCO_3_^−^], arterial lactate, and urine output> 0.5 mL/kg/h. These covariates were selected for the result of the bivariate analysis of each one of them with the independent and dependent variables. However, only some of these significant covariates were chosen for propensity score matching. For the estimation of the coefficients by Logistic Regression to be stable, one predictor for every 10–20 cases with the event or non-event (pH ≤ 7.28 or > 7.28), whichever is lower, must be introduced into the model [9].

In the file paired with PSM there are 135 cases with pH ≤ 7.28 and 140 cases with pH > 7.28. Therefore, for the model to be stable, between 6.5 and 13 predictors can be introduced, and in our study, we have introduced 9 predictor variables. The quantitative selection of the predictors to include in the model has therefore been carried out with a statistical criterion.

For the qualitative selection of the predictors to be introduced in the model, a scientific criterion is used. Those variables with a significant result in the bivariate analysis were included that also influence the response due to theoretical knowledge, excluding those correlated with others already present because they produce collinearity problems [10]. *P*-values for multiple comparisons are corrected by Holm's multiple test procedure [11].

A p-value of ≤ 0.05 was considered significant. All statistical analysis was performed using IBM SPSS Statistics 25.00 (IBM Corp., Armonk, NY, USA).

### Role of the funding source

The funders of the study had no role in study design, data collection, data analysis, data interpretation, or writing of this paper.

## Results

Two hundred ninety-seven patients monitored by the PiCCO™ who met the inclusion criteria were recruited into the study. In most patients (*N* = 162; 54.5%), PiCCO™ monitoring was established due to the presence of arterial hypotension related to cardiac dysfunction and/ or sepsis.

This was a prospective multicenter observational cross-sectional study. As data were collected from six ICU's of different hospitals, in the Appendix (Tablets 4 and 5) are described the sample size by each ICU involved in the study after PSM and the linear regression between pH and the six dependent variables of cardiac power index (W/m^2^) adjusted by Propensity Score Matching in the six ICUs.

The median age of the patients was 59 (IQR 49–71) years, 150 (50.5%) of the patients were male. The demographics and clinical characteristics of the patients are summarized in Table 1. Acid-base parameters, and pertinent electrolytes are shown in Table 2 and therapeutic measures in Table 3. All patients had undergone intubation and were under controlled mechanical ventilation while receiving vasopressor and/or inotropic support as necessary Table 3. Among the patients’ demographics and clinic characteristics, there were significant difference in the distribution of sex (male were 56.9% at pH ≤ 7.28 and 43.8% at pH > 7.28; *p* = 0.02), death at day 28 (60.3% at pH ≤ 7.28 and 25.4% at pH > 7.28; *p* < 0.001), ischemic heart disease (13.7% at pH ≤ 7.28 and 23.6% at pH > 7.28; *p* = 0.02), cardiac surgery (0.7% at pH ≤ 7.28 and 6,3% at pH > 7.28; *p* = 0.009), septic shock (medical) (19.6% at pH ≤ 7.28 and 9.0% at pH > 7.28; *p* = 0.01) and trauma/neurosurgery (4.6% at pH ≤ 7.28 and 16,0% at pH > 7.28; *p* = 0.001) between pH categories. There were significant differences in APACHE II (median 23 at pH ≤ 7.28 and 19 at pH > 7.28; *p* < 0.001) and constant urine output > 0.5 mL/kg/h (15.9% at pH ≤ 7.28 and 45.1% at pH > 7.28; *p* < 0.001) between pH categories. Among constants, body temperature (median 36.5 °C at pH ≤ 7.28 and 36.8 °C at pH > 7.28; *p* = 0.001), heart rate (median 104 bpm at pH ≤ 7.28 and 88 at pH > 7.28; *p*<0.001), systolic blood pressure (median 108 mmHg at pH ≤ 7.28 and 115 at pH > 7.28; *p* = 0.001), diastolic blood pressure (median 53 mmHg at pH ≤ 7.28 and 57 at pH > 7.28; *p*<0.001), mean blood pressure (median 68 mmHg at pH ≤ 7.28 and 76 at pH > 7.28; *p* < 0.001) and central venous pressure (median 14 mmHg at pH ≤ 7.28 and 13 at pH > 7.28; *p* = 0.03) were significantly different between pH categories. Furthermore, inspiratory oxygen fraction (45% at pH ≤ 7.28 and 35% at pH > 7.28; *p* < 0.001), oxygen saturation (96% at pH ≤ 7.28 and 97% at pH > 7.28; *p* = 0.01), arterial CO_2_ tension (median 6.0 kPa at pH ≤ 7.28 and 5.6 at pH > 7.28; *p* < 0.001), bicarbonate (median 17.5 mmol/L at pH ≤ 7.28 and 22.5 at pH > 7.28; *p* < 0.001), bases excess (median −8.5 mmol/L at pH ≤ 7.28 and −2.2 at pH > 7.28; *p* < 0.001) and arterial O_2_-saturation (95.0% at pH ≤ 7.28 and 96.3% at pH > 7.28; *p* < 0.001) were significantly different between pH categories. Among laboratory values, potassium (median 4.6 mmol/L at pH ≤ 7.28 and 4.4 at pH > 7.28; *p* = 0.03), ionized calcium (median 1.17 mmol/L at pH ≤ 7.28 and 1.18 at pH > 7.28; *p* = 0.03), blood glucose (median 7.6 mmol/L at pH ≤ 7.28 and 8.3 at pH > 7.28; *p* = 0.05), lactate (median 4.5 mmol/L at pH ≤ 7.28 and 1.6 at pH > 7.28; *p* < 0.001), plasma total calcium (median 2.03 mmol/L at pH ≤ 7.28 and 2.06 at pH > 7.28; *p* = 0.05), phosphate (median 1.49 mmol/L at pH ≤ 7.28 and 1.16 at pH > 7.28; *p* < 0.001) and total protein (median 48 g/L at pH ≤ 7.28 and 50 at pH > 7.28; *p* = 0.03) were significantly different between pH categories. Among therapeutic measures, sedation level (RASS) (median −4 at pH ≤ 7.28 and −4 at pH > 7.28; *p* = 0.01), red cell (51% at pH ≤ 7.28 and 31% at pH > 7.28; *p* = 0.02) and fresh frozen plasma (16.3% at pH ≤ 7.28 and 6.3% at pH > 7.28; *p* = 0.01) were significantly different between pH categories. There were significant differences in the distribution of noradrenaline (55.9% > 0.5 µg/kg/min at pH ≤ 7.28 and 20.4% at pH > 7.28; *p* < 0.001), vasopressin/terlipressin (21.6% > 0.02 units/min at pH ≤ 7.28 and 7.7% at pH > 7.28; *p* = 0.001), bicarbonate infusion (3.9% > 42 ng/mL at pH ≤ 7.28 and 0.7% at pH > 7.28; *p* < 0.001) and crystalloids (24.2% > 2000 mL at pH ≤ 7.28 and 10.5% at pH > 7.28; *p* = 0.02) between pH categories.

**Table 3:** Therapeutic measures of pH categories ≤ 7.28 and > 7.28.

	pH ≤ 7.28 n (%) or Md (IQR)*	pH > 7.28 n (%) or Md (IQR)*	*P*-value
Sedation level (RASS)	−4 (−5 - −4)	−4 (−5– −3)	0.01
Inotropic support (hours)	24 (9 - 45)	19 (10 - 36)	0.11
Noradrenaline			< 0.001
> 0.5 µg/kg/min	85 (55.9)	29 (20.4)	
< 0.5 µg/kg/min	60 (39.5)	91 (64.1)	
Dobutamine			0.12
> 0.5 µg/kg/min	11 (7.2)	3 (2.1)	
< 0.5 µg/kg/min	3 (2.0)	2 (1.4)	
Milrinone			> 0.20
> 0.25 µg/kg/min	28 (18.3)	19 (13.3)	
< 0.25 µg/kg/min	12 (7.8)	10 (7.0)	
Adrenaline			> 0.20
> 0.20 µg/kg/min	7 (4.6)	2 (1.4)	
< 0.20 µg/kg/min	5 (3.3)	3 (2.1)	
Vasopressin/Terlipressin			0.001
> 0.02 units/min	33 (21.6)	11 (7.7)	
< 0.02 units/min	18 (11.8)	10 (7.0)	
CRRT (default mode CVVHDF) < 36 mL/kg	87 (37.5)	44 (30.8)	> 0.20
CRRT (default mode CVVHDF) > 36 mL/kg	67 (44.4)	21 (14.7)	< 0.001
Bicarbonate infusion			< 0.001
> 42 ng/mL	6 (3.9)	1 (0.7)	
6.4 - 42 ng/mL	16 (10.5)	1 (0.7)	
< 6.4 ng/mL	7 (4.5)	1 (0.7)	
Crystalloids			0.02
> 2000 mL	37 (24.2)	15 (10.5)	
1000 – 2000 mL	28 (18.3)	32 (22.4)	
<1000 mL	46 (30.1)	46 (32.2)	
Red cell	51 (33.3)	31 (21.5)	0.02
Fresh frozen plasma	25 (16.3)	9 (6.3)	0.01
Platelets	21 (13.7)	12 (8.3)	0.14
Albumin			0.18
> 120 g/L	5 (3.3)	2 (1.4)	
60 - 120 g/L	15 (9.8)	6 (4.2)	
< 60 g/L	22 (14.4)	21 (14.6)	

*Values are frequency count (N) and percent (%) for categorical variables or median (Md) and Interquartile range (IQR) for quantitative variables.

**Fig. 2:**
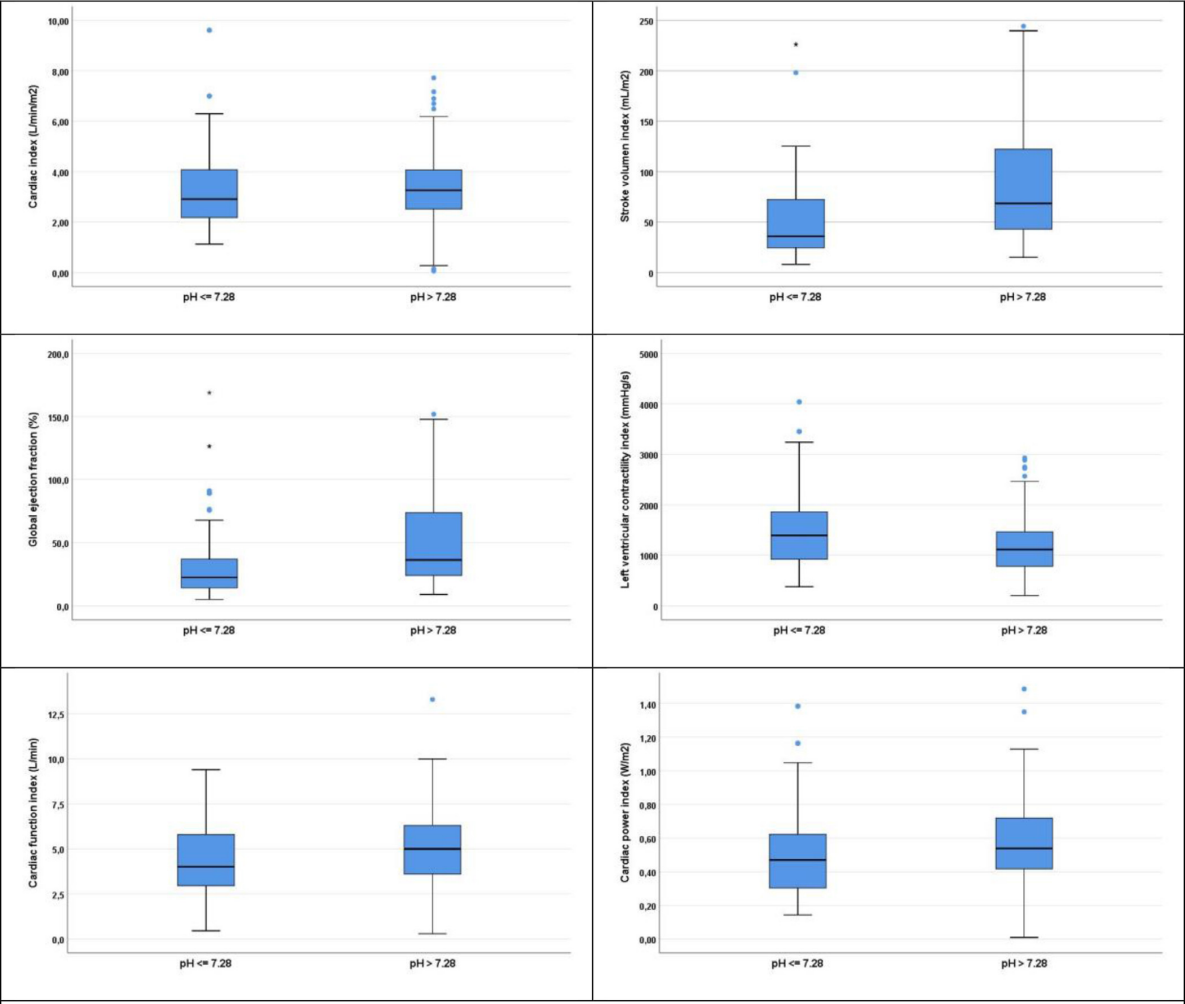
**Boxplots of cardiac function variables in categories of pH ≤ 7.28 and > 7.28.** Boxplots present the adjusted relationships graphically. In all plots, except that for LVCI, midline within the box (median) and limits of boxes (IQR) of the group with pH ≤ 7.28 were lower than those of the group with pH > 7.28.

After propensity score matching the 9 variables used for adjusting were more equilibrated between groups as seen in Table 4 and in the histograms before and after adjusting by PSM (see Figs. 1 and 2 of APACHE in the appendix).

**Table 4:** Predictors variables of pH groups ≤ 7.28 and > 7.28 after propensity score matching.

	pH ≤ 7.28 n (%) or Md (IQR)*	pH > 7.28 n (%) or Md (IQR)*	*P*-value
Sex Male	76 (56.3)	62 (44.3)	0.061
APACHE II score	20 (17–24)	19 (16–23)	0.089
Constant urine output > 0.5 mL/kg/h	42 (31.1)	60 (42.9)	0.059
Body temperature ( °C)	36.6 (35.5–37.7)	36.8 (36.4–37.6)	0.061
Systolic blood pressure (mmHg)	114 (106–123)	118 (110–126)	0.084
Arterial CO_2_ tension (kPa)	5,7 (5,1–6,4)	5,6 (5,1–6,0)	0.111
Bicarbonate (mmol/L)	22.1 (20.2–23.4)	22.6 (21.0–24.6)	0.114
Lactate (mmol/L)	3.8 (2.7–5.0)	2.8 (2.1–4.6)	0.112
Noradrenaline	127 (94.1)	120 (85.7)	0.036

*Values are frequency count (N) and percent (%) for categorical variables or median (Md) and interquartile range (IQR) for quantitative variables.

**Fig. 3:**
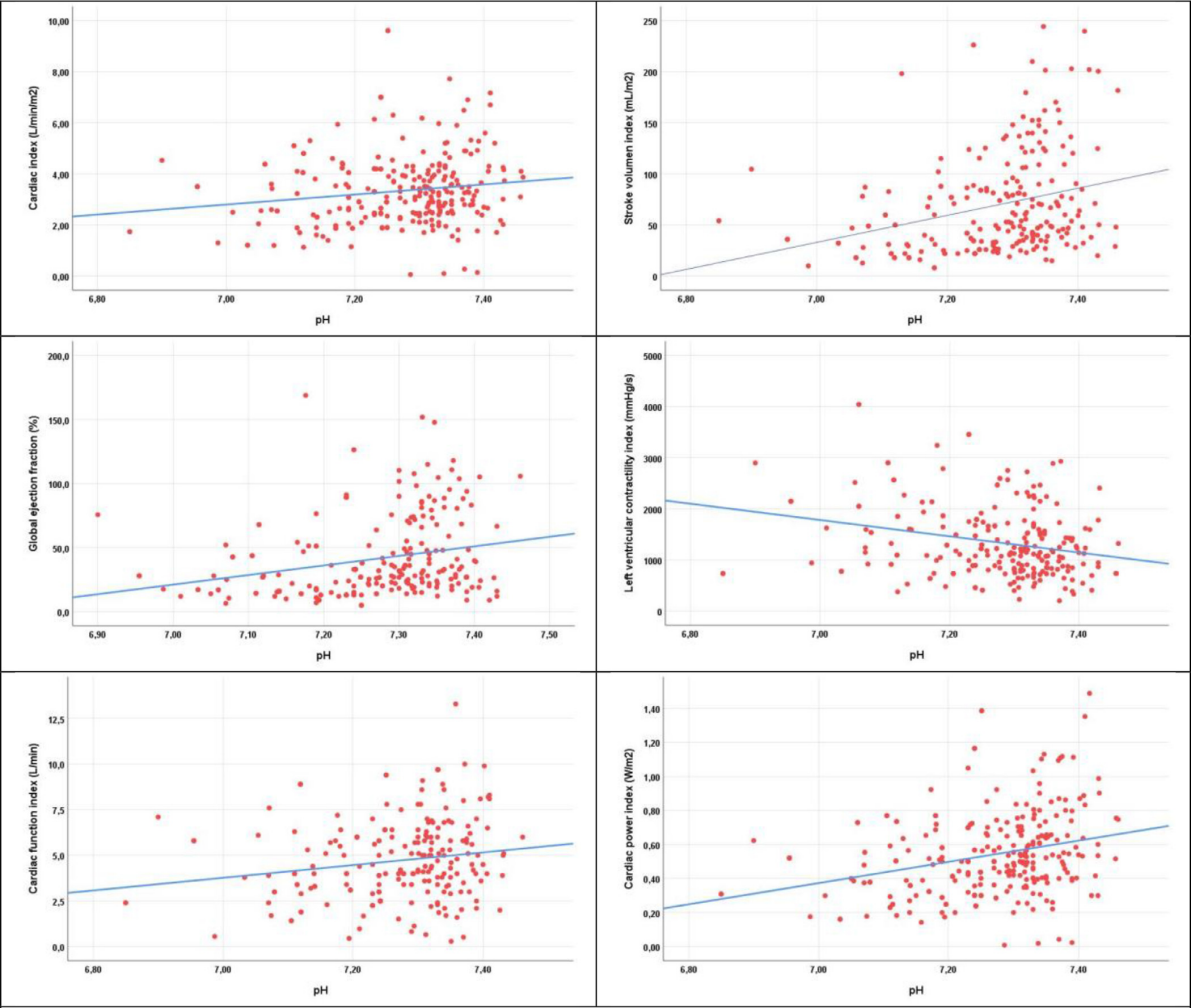
**Scatterplots between cardiac function variables and pH.** The regression line is indicated in blue. Scatterplots with regression lines present these 6 relationships graphically. Five of the regression lines are ascendant, which means that the correlation is positive, as cardiac function increases with pH increasing (For interpretation of the references to color in this figure legend, the reader is referred to the web version of this article.).

### The effect of arterial pH on cardiac function

The cardiac function in the two pH categories and the results of simple linear regression with cardiac function as the dependent variable and dichotomized pH (cut-off 7.28) as independent variable are shown in Table 5. In addition, similar simple regression analysis was performed using quantitative arterial pH as the independent variable (Table 6). To control for potential confounders, similar analysis was performed with Propensity Score Matched sample (Tables 7 and 8).

**Table 5:** Unadjusted simple linear regression between pH ≤ 7.28 and > 7.28 and the dependent variables of cardiac function. There is a significant difference in all measures of cardiac function between the two categories except for cardiac index.

	pH ≤ 7.28 Md (IQR)	pH > 7.28 Md (IQR)	Difference in means (pH> 7.28 vs. pH ≤ 7.28, 95% CI)	*P*- value
N (%)	153 (51.5)	144 (48,5)		
Cardiac index (L/min/m^2^)	3.07 (2.29–4.15)	3.27 (2.52–4.07)	0.01 (−0.33–0.35)	0.26
Stroke volume index (mL/m^2^)	42 (26–82)	69 (43–122)	24 (11–36)	< 0.001
Global ejection fraction (%)	25.0 (16.0–51.6)	36.4 (24.1–73.8)	10.4 (1.7–19.2)	< 0.001
dPmax (mmHg/s)	1393 (1039–1792)	1116 (783–1465)	−280 (−432– −128)	< 0.001
Cardiac function index (L/min)	4.0 (3.0–5.6)	5.0 (3.6–6.3)	0,.3 (0.1–1.2)	0.02
Cardiac power index (W/m^2^)	0.46 (0.32–0.65)	0.54 (0.42–0.72)	0.06 (0.00–0.12)	0.004

**Table 6:** Unadjusted simple linear regression between pH and the six dependent variables of cardiac function (*n* = 297 cases).

	Regression Coefficient	95% CI Regression Coefficient	*P*-value
Cardiac index (L/mim/m^2^)	1.35	0.03–2.66	0.05
Stroke volume index (mL/m^2^)	41	−8–89	0.10
Global ejection fraction (%)	18.6	−15.2–52.3	> 0.20
dPmax (mmHg/s)	−1002	−1581– −424	0.001
Cardiac function index (L/min)	2.01	−0.13–4.15	0.07
Cardiac power index (W/m^2^)	0.45	0.22–0.69	< 0.001

Among the six cardiac function measures, pH was significantly associated with CI, dPmax and CPI, respectively.

**Table 7:** Linear regression between pH ≤ 7.28 and pH > 728 and the six dependent variables of cardiac function adjusted by propensity score matching.

	pH ≤ 7.28 Md (IQR)	pH > 7.28 Md (IQR)	Difference in mean (pH > 7.28 vs. pH ≤ 7.28, 95% CI)	*P*- value corrected
N (%)	135 (49.1)	140 (50.9)		
Cardiac index (L/min/m^2^)	2.91 (2.18–4.08)	3.27 (2.52–4.07)	0.13 (−0.20–0.47)	0.115
Stroke volume index (mL/m^2^)	36 (25–73)	69 (43–122)	33 (21–45)	< 0.001
Global ejection fraction (%)	22.6 (14.2–37.1)	36.4 (24.1–73.8)	18.3 (10.5–26.0)	< 0.001
dPmax (mmHg/s)	1397 (922–1863)	1116 (783–1465)	−331 (−510– −153)	0.003
Cardiac function index (L/min)	4.0 (3.0–5.8)	5.0 (3.6–6.3)	0.7 (0.2–1.3)	0.024
Cardiac power index (W/m^2^)	0.47 (0.30–0.62)	0.54 (0.42–0.72)	0.09 (0.03–0.15)	0.002

The significant difference in means (values for the category pH > 7.28 minus those in category pH ≤ 7.28) for the five measures of cardiac function measures are: Stroke volume index 33 (95% CI 21 to 45) mL/m^2^ (*p* < 0.001); Global ejection fraction 18.3(95% CI 10.5 to 26.0% (*p* < 0.001); dPmax −331 (95% CI −510 to −153) mmHg/s (*p* = 0.003); Cardiac function index 0.7 (95% CI 0.2 to 1.3) L/min (*p* = 0.024); Cardiac power index 0,09 (95% CI 0.03 to 0.15) W/m^2^ (*p* = 0.002); Cardiac index (0.13; 95% CI −0.20 to 0.47 L/min/m^2^; *p* = 0.12) was not significantly different between pH categories.

**Table 8:** Linear regression between pH and the six dependent variables of cardiac function adjusted by propensity score matching.

	Regression coefficient	95% CI Regression coefficient	*P*- value corrected
Cardiac index (L/min/m^2^)	1.96	0.45–3.47	0.011
Stroke volume index (mL/m^2^)	132	77–188	< 0.001
Global ejection fraction (%)	74.7	37.1–112.4	< 0.001
dPmax (mmHg/s)	−1587	−2361–−815	< 0.001
Cardiac function index (L/min)	3.47	0.87–6.07	0.018
Cardiac power index (W/m^2^)	0.62	0.36–0.88	< 0.001

In the adjusted analysis, all six measures of cardiac function were affected by arterial pH. The regression coefficients were: Cardiac index 1.96 (95% CI 0.45 to 3.47) L/min/m^2^ (*p* = 0.011). For each unit of pH increase, cardiac index increased by 1.96 L/min/m^2;^ Stroke volume index 132 (95% CI 77 to 188) mL/m^2^ (*p* < 0.001). For each unit of pH increase, stroke volume index increased by 132 mL/m^2;^ Global ejection fraction 74.7 (95% CI 37.1 to 112.4)%; (*p* < 0.001). For each unit of pH increase, global ejection fraction increased by 74.7%; dPmax −1587 (95% CI −2361 to −815) mmHg/s; (*p* < 0.001). For each unit of pH increase, dPmax decreased by 1587 mmHg/s; Cardiac function index 3.47 (95% CI 0.87 to 6.07) L/min (*p* = 0.018). For each unit of pH increase, cardiac function index increased by 3.47 L/min; Cardiac power index 0.62 (95% CI 0.36 to 0.88 W/m^2^ (*p* < 0.001). For each unit of pH increase, cardiac power index increased by 0.62 W/m^2^.

Table 6 shows the significant difference in means (values for the category pH > 7.28 minus those in category pH ≤ 7.28) for the five measures of cardiac function measures are: Stroke volume index 33 (95% CI 21 to 45) mL/m^2^ (*p* < 0.001); Global ejection fraction 18.3(95% CI 10.5 to 26.0% (*p* < 0.001); dPmax −331 (95% CI −510 to −153) mmHg/s (*p* = 0.003); Cardiac function index 0.7 (95% CI 0.2 to 1.3) L/min (*p* = 0.024); Cardiac power index 0,09 (95% CI 0.03 to 0.15) W/m^2^ (*p* = 0.002); Cardiac index (0.13; 95% CI −0.20 to 0.47 L/min/m^2^; *p* = 0.12) was not significantly different between pH categories.

Table 7 shows the regression coefficients were: Cardiac index 1.96 (95% CI 0.45 to 3.47) L/min/m^2^ (*p* = 0.011). For each unit of pH increase, cardiac index increased by 1.96 L/min/m^2^; Stroke volume index 132 (95% CI 77 to 188) mL/m^2^ (*p* < 0.001). For each unit of pH increase, stroke volume index increased by 132 mL/m^2^; Global ejection fraction 74.7 (95% CI 37.1 to 112.4)%; (*p* < 0.001). For each unit of pH increase, global ejection fraction increased by 74.7%; dPmax −1587 (95% CI −2361 to −815) mmHg/s; (*p* < 0.001). For each unit of pH increase, dPmax decreased by 1587 mmHg/s; Cardiac function index 3.47 (95% CI 0.87 to 6.07) L/min (*p* = 0.018). For each unit of pH increase, cardiac function index increased by 3.47 L/min; Cardiac power index 0.62 (95% CI 0.36 to 0.88 W/m^2^ (*p* < 0.001). For each unit of pH increase, cardiac power index increased by 0.62 W/m^2^

Based on these results, a reduced arterial pH was associated with impaired cardiac function and contractility.

## Discussion

A reduction in cardiac output and blood pressure with a resultant decrease in perfusion of vital tissues is common in seriously ill patients hospitalized in the ICU. These physiological abnormalities are considered important factors contributing to a high mortality in these patients. Indeed, a primary decrease in cardiac function or failure of an adaptive increase in cardiac function in response to injurious events is postulated to play a dominant role. Direct damage to the myocardium or hormonal derangements are potential factors in causing myocardial dysfunction. However, it is unclear as to what other factors might contribute. Previous studies in normal dogs showed that cardiac function was impaired when the systemic pH fell finding consistent with the hypothesis that the acidemia can contribute to abnormal cardiac function by measuring the left ventricular diastolic volume (LFDV) and left ventricular end-systolic pressure-volume relation (ESPVR) [4], [5]. Despite these observations obtained more than 40 years ago in animals, only a single letter to the Editor in the New England Journal of Medicine examined the relationship between acidemia and cardiac function. In this study, it was revealed that fractional shortening of the left ventricle during systole in 10 consecutive patients (6 women and 4 men; mean [±SD] age, 42±18 years), 7 of whom were admitted for treatment of diabetic ketoacidosis and 3 of whom were admitted for alcoholic ketoacidosis was not different during the period of acidemia compared to the period after its spontaneous resolution [6]. However, no studies of a larger cohort of patients have examined the effect of systemic acidemia on cardiac function in seriously ill humans hospitalized in the ICU.

Therefore, in the present study we examined the relationship between systemic blood acidity and cardiac function in a large cohort of seriously ill patients hospitalized in several ICUs in London, England.

To obtain accurate measurements of cardiac function we used the transpulmonary thermodilution technique with integrated pulse contour analysis under rigorous inclusion criteria. This system provides intermittent (transpulmonary thermodilution-derived, TPTD) and continuous (pulse contour-derived, PCD) assessment of cardiac output and estimations of intrathoracic volumes, cardiac output and left ventricular systolic function). This system has been shown to give an accurate estimation of cardiac output and left ventricular systolic function [7,8,[12], [13], [14], [15], [16], [17]] with specific cardiovascular variables for the measurement of cardiac contractility such the GEF and the CFI [16] among other cardiovascular parameters never used before in the context of acidemia [8,13,[18], [19], [20], [21], [22], [23], [24], [25]]. The TPTD technique is a less invasive alternative than the pulmonary artery catheter [26], [27] which relative easily provides measurements of cardiac function at the bedside. Similar information may be obtained by echocardiography as well, but this technique requires an even more experienced examiner and ideally the same certified examiner for all the centers [16,17]. Direct comparation with previous experimental animal studies reported in the past is not possible for very relevant reasons. First, in the previous experimental studies performed in dogs in a controlled environment, cardiac function was measured during progressive titration of systemic pH levels below 7.00 and was shown to be depressed as systemic pH fell. However, in the present observational study, examining cardiac function using the PiCCO ™ technique at comparable levels of mentioned acidemia was not considered ethically acceptable. Although many of the critically ill patients were profoundly acidemic before or in the early period after admission to the ICU, recruiting these patients at the point of severe acidemia into the study was extremely difficult.

Indeed, by the time the patient is referred to our ICU service from emergency department (ED) and admitted in ICU, a central access is obtained and the cardiovascular monitoring is established and calibrated, and the patient intubated and mechanically ventilated, the plasma pH had already increased the patient having received in many occasions fluids, hemoderivates and also inotropic support to maintain arterial blood pressure following the basic rules in the approach and management of the critically ill patient. Therefore, periods of severe acidemia were not always available. Based on the data available to us, a cut-off plasm pH of 7.28 was found to divide patients into two equal groups (pH ≤ 7.28, *N* = 153 vs pH > 7.28, *N* = 144). Comparing these groups, there was a significant for the following variables of cardiac function: SVI (difference in means 32.7; 95% CI: 21 to 45 mL/m^2^; *p* <0.001); GEF (18; 95% CI: 11 to 26%; *p* < 0.001), dPmax (−331; 95% CI: −510 to −153 mmHg/s; *p* = 0.001), CFI (0.7; 95% CI: 0.2 to 1.3 1/min; *p* = 0.01) and CPI (0.09; 95% CI: 0.03 to 0.15 W/m^2^; *p* < 0.001). Also, simple linear regression analysis of cardiac function and blood pH in all patients showed that cardiac function was depressed with acidemia.

These results differ somewhat from studies done in animalsfor various reasons. The experimental protocol is significantly different. In experiments in which the thorax was opened, a direct canulations was performed and an ultrasound was use in isolated myocardium to measure the left ventricular volume. Thoracotomy and pericardiotomy alter the diastolic pressure-volume relation by mechanical interaction of the heart, pericardium, and lungs venous return is influenced by diastolic pressures and conceivably would be different in a closed-chest model. Also, in the whole-animal studies indexes of contractility can be confounded by changes in preload, afterload, and heart rate [28].

In a canine study, on isolated hearts, lactic acidosis caused a 40% reduction in stroke volume, which could be attributed to depressed LV contractility potentially due to increase end-diastolic volume as a result of acute pulmonary hypertension [5]. In another similar study, respiratory acidosis showed a decreased LV contractility and a fall in SVR, however cardiac output was described to have increased associated with a similar increase in heart rate. This increment was ascribed to an outpouring of catecholamines into the circulation by the stimulus of acidemia, since it could be blocked by administration of β-blockers [29]. Another study in lambs confirmed these results [30]. In studies performed during early development in lambs have shown that the responses elicited after chemostimulation with veratridine are dependent on the age of the animal, although in this study vagotomy significantly attenuates the depressor reflex caused by hypotension, it did not modify the excitatory response to veratridine in near-term fetal lambs, suggesting that the response to veratridine was not vagally mediated. In fact, the excitatory response to veratridine was inhibited by propranolol, suggesting again, a sympathetic-mediated response or a direct effect of veratridine on the release of catecholamines resulting in a paradoxical increase in HR and blood pressure in preterm fetal lambs and eliciting the classic depressor reflex (hypotension and bradycardia), Bezold-Jarisch reflex (BJR) in term lambs [31]. The studies performed in fetal sheep are one of the few models where acidemia has been studied extensively due to the focus on the effects of acidemia on fetal brain during the trial of labour. It has been shown in this model that fetal arterial blood pressure and heart rate does respond to progressive acidemia. During prolonged partial cord occlusion, fetal heart rate (FHR) decreased initially, then recovered to above control value; this occurred in the face of a significant acidosis [32]. These conclusions are compatible with previous studies in dogs where the hearts of intact animals with normal sympathoadrenal responses will develop a compensatory response, beta-receptor stimulation during acidosis results from increased release of norepinephrine from cardiac nerve endings and epinephrine and norepinephrine from the adrenal medulla. In our study we observed similar results, with lower pH using a cut-off value of 7.28, a significantly lower stroke volume associated with an inadequate increase in heart rate for maintenance of cardiac output as well as a reduction in GEF, CFI and CPI. Only dPmax regarded as a marker of left ventricular contractility was found to have an inverse relationship, it increased in presence of acidosis. However, dPmax is merely then a marker of cardiac contractility, when changes in heart rate, left ventricular end-diastolic volume and aortic pressure (outflow impedance is the most important part of cardiac afterload) are considered. As for those factors no correction or estimation was made, interpretation of this finding is limited and must remain open. On the other hand, loss in vascular resistance (SVRI) during acidemia as one factor may lead to an increase in dPmax and most of our patients with low pH were on noradrenaline. There were significant differences between pH categories in supporting this interpretation of noradrenaline (*p* < 0.001), vasopressin/terlipressin (*p* = 0.001) and blood pressure.

In an animal study, Morimont et al. compared arterial dPmax with end-systolic elastance (Ees), the gold standard method for assessing LV contractility. These authors described that endotoxin-induced shock and catecholamine infusion lead to significant variations in LV contractility. Despite statistical significance (*r* = 0.51; *p* < 0.001) which at best can be classified as moderate, low agreement between the two methods were observed. However, a far better correlation with a good agreement were observed when positive-pressure ventilation induced an arterial pulse pressure variation (PPV) of ≤ 11% (*r* = 0.77; *p* < 0.001) [26]. In our population, PPV on average was 11% in the low pH category of ≤ 7.28 with a relatively wide scatter (25.–75. percentile 7–23%) which may limit the interpretation. (Table 3; Appendix)

In conclusion, our results show that systemic acidemia is associated with impairment of several measures of cardiac function. Furthermore, a significant relationship was found between the quantitative pH and the degree of cardiac function, this finding is common with previous experimental studies where a progressive fall in pH during acid infusion in experimental models is associated with a decrease in cardiac output. These data support the potential value of a early detection of acidemia [33,34] and the possible benefit of improving arterial pH in these patients.

In a previous randomized controlled trial in ICU patients with severe metabolic acidemia, treatment with sodium bicarbonate had no effect on the primary composite outcome (death from any cause by day 28 and the presence of at least one organ failure at day 7) [35] despite improvement in systemic pH. However, no measures of cardiac function were available in that study.to determine if base therapy improved or worsened cardiac function. Further rigorously performed prospective randomly controlled studies to examine the benefits and limitations of different methods of treating systemic acidemia in seriously ill patients are warranted.

## Declaration of Competing Interest

Jeffrey A. Kraut's has a pending patent in association with Tom Mason (UCLA Biochemistry) on “New Base in Treatment of Metabolic Acidosis” and reports payment from the American Society of Nephrology “Kidney Week 2018”. Samir G. Sakka is a member of the Medical Advisory Board of Pulsion, Maquet Getinge Group; All rest of faculty and staff in a position to control or affect the content of this paper have declared that they have no competing financial interests or institutional conflicts. The rest of authors have declared that no competing interests exist.

## Funding

These studies were supported in part by unrestricted funds from University of California at Los Angeles (JAK).

## Data sharing statement

In accordance with the Declaration of Helsinki regarding the confidentiality of the patient's information, The General Data Protection Regulation (25 May 2018), local applicable regulatory requirements and the ethical approvals for the study, the de identified study data can be made available to researchers upon request and after approval by the local Research and Development Department and the Regional Ethics committee for researchers who meet the criteria for access to confidential data.

## Appendix Supplementary materials

Image, application 1

Image, application 2
