# Supplementary material for: Systemic acidemia impairs cardiac function in critically Ill patients
Source: eClinicalMedicine. 2021 Jun 29;37:100956. doi: 10.1016/j.eclinm.2021.100956 (PMC8255172; doi:10.1016/j.eclinm.2021.100956)
Supplement: Supplementary file 1 [file mmc1.docx]

**Table 1: Inclusion and exclusion criteria**

**Inclusion criteria**

- Age >18 years and PiCCO™ monitoring according to clinical decision by the consultant responsible in each intensive care unit
- Only NHS hospitals
- Mechanical ventilation with a tidal volume of 6-8 ml/kg

**Exclusion criteria**

- NO to include vulnerable adults lacking capacity to consent for themselves.
- NO to include any participants who are prisoners or young offenders in the custody of HM Prison Service or who are offenders supervised by the probation service in England.
- Atrial or ventricular arrhythmia: a stable pulse is required for reliable pulse contour analysis.
- Pre-known aortic, mitral or tricuspid valve insufficiency. In the case of valve insufficiency, the valve does not close correctly. Therefore, the thermodilution curve is affected by indicator regurgitation, resulting in a prolonged indicator decay time.
- Pre-known ascending aorta pathologies. Intra-Aortic Balloon pump:   pulse contour analysis is invalid; transpulmonary thermodilution still valid for cardiac output measurements.
- Pre-known aortic aneurysm: pulse contour analysis will be bizarre because the arterial compliance is going to be weird, with the aortic aneurysm acting as a damping system by absorbing all the pressure wave.
- Extracorporeal circuit: when on bypass, there is no real arterial pressure waveform.
- Pneumonectomy: PICCO™ relies on a relatively normal pulmonary vasculature.
- Massive pulmonary embolism: see pneumonectomy.
- Intracardiac shunt:  transpulmonary thermodilution will give an inaccurate reading.
- A tidal volume of less than 6-8mL/kg.
- NON-positive pressure ventilated patients. Severe obesity.
- Open abdomen.

**Table 2: Normal values and ranges of haemodynamic parameters.**

**Thermodilution Parameters**

- **Cardiac output:**
- CO-cardiac output: 4 -8 L/min.^(a)^
- CI-cardiac index: 3- 5 L/min/m^₂^.^(a)^
- **Preload:**
- GEDVI-global end-diastolic volume index: 680- 800 mL/m^₂^.^(a)^
- ITBVI-intrathoracic blood volume index: 850-1000 mL/m^₂^.^(a)^
- **Pulmonary oedema:**
- ELWI-extravascular lung water index: 3-7 mL/kg ^(a)^
- PVPI-pulmonary vascular permeability index: 1.0- 3.0 (1-3 cardiogenic oedema; > 3 permeability oedema) ^(a)^
- **Contractility:**
- CFI-cardiac function index: 4.5- 6.5 L/min ^(a)^
- GEF-global ejection fraction: 25- 35% ^(a)^

**Pulse contour Parameters**

- **Flow**
- PCCI-pulse contour cardiac output index: 3.0-5.0 L/min/m^₂^.^(b)^
- ABP-arterial blood pressure mmHg.^(b)^
- HR-heart rate 1/min.^(b)^
- SVI-stroke volume index: 50-110 ml.^(b)^
- **Volume responsiveness:**
- SVV-stroke volume variation: <10% ^(b)^
- PPV-pulse pressure variation: <10% ^(b)^
- **Afterload:**
- SVRI-systemic vascular resistance index: 1700-2400 dyn*s*cm-5*m^2^.^(b)^
- **Contractility:**
- CPI- cardiac power index 0.5-0.7 W/m^2^.^(b)^
- dPmax - steepness of the upward part of arterial pressure curve :1000-1200mmHg/s. ^(b)^1200 mmHg/s

(a) These parameters can only be obtained intermittently by performing a TPTD as calibration.

(b) Parameters calculated from the arterial pulse contour on a continuous basis.

**CO** (Cardiac Output) = SV (Stroke Volume) x HR (Heart Rate). **CI** (Cardiac Index) = SVI (Stroke Volume Index) x HR (Heart Rate). **GEDVI** (Global End-Diastolic Volume Index) = GEDV (Global End-Diastolic Volume)/m^2^. GEDV: it is the volume of blood contained in the 4 chambers of the heart. ITBV: it is the volume of the 4 chambers of the heart plus the blood volume in the pulmonary vessels. **ITBVI** (Intra Thoracic Blood Volume Index) **=** ITBV (Intra thoracic blood volume) /m^2^. EVLW: amount of water content in the lungs or degree of pulmonary oedema. **ELWI** (Extravascular Lung Water Index) = ELW (Extravascular Lung Water) / m^2^). **PVPI**: indication of pulmonary oedema in relation to preload. **CFI** (Cardiac function index) **=** CI (Cardiac index)/ GEDV (Global End Diastolic Volume). CFI: ratio of the index of cardiac output to the index of the GEDV. A measure of how well the CO is doing in relation to its preload. **GEF**: % of total blood expelled from the heart every beat to the total amount of blood estimated to be present just prior to ventricular systole. GEF (Global ejection fraction) **=** [4 X SV (Stroke Volume)]/GEDV (Global End Diastolic Volume). **SVV**: reflects the sensitivity of the heart to the cyclic changes in cardiac preload induced by mechanical ventilation. Can predict whether stroke volume will increase with volume expansion. SVV in %= 100 X (SV _max_-SV _min_)/(SV _mean_) over a period of time (12 seconds). **PPV** in %= 100 X (PP _max_-PP_min_)/(PP_mean_). **SVRI** (Systemic Vascular Resistance Index) = 80 X [MAP(Mean Arterial Pressure)–CVP(Central Venous Pressure)]/CI (Cardiac Index). **CPI** represents the power of left ventricular cardiac output in watts. CPI (cardiac power index) = MAP × CI/451. **dPmax**-. The dPmax is a measure of how fast the pressure rises during systole and is regarded as a marker of left ventricular contractility.

| **Table 3: Haemodynamic parameters of pH categories ≤ 7.28 and > 7.28** | | | |
| --- | --- | --- | --- |
|  | **pH** ≤ **7,28**  Md (IQR)* | **pH > 7,28**  Md (IQR)* | **p value** |
| ITBVI (Intra Thoracic Blood Volume Index) mL/m^₂^ | 826  (697 – 984) | 859  (715 - 1054) | 0.31 |
| GEDI (Global End-Diastolic volume Index) mL/m^₂^ | 668  (563 -785) | 688  (569 - 876) | 0.36 |
| ELWI (Extravascular Lung Water Index) mL/kg | 10.0  (7.7 – 14.0) | 8.5  (6.9 - 12.0) | 0.01 |
| PVPI (Pulmonary Vascular Permeability Index) | 2.4  (1.7 – 3.2) | 1.8  (1.6 – 2.5) | 0.09 |
| SVV (Stroke Volume Variation) % | 15  (9 -22) | 11  (6 -17) | < 0.001 |
| PPV (Pulse Pressure Variation) % | 11  (7 -23) | 8  (6 - 13) | 0.42 |
| SVRI (Systemic Vascular Resistance Index) dyn*s*cm^-5^*m^2^ | 1573  (1054 - 1906) | 1546  (1194 - 1943) | 0.71 |
| *Values are median (Md) and Interquartile range (IQR) | | | |

| **Table 4**. Sample size by each ICU involved in the study after PSM. | | |
| --- | --- | --- |
| **ICU** | **N** | **%** |
| LIVER | 71 | 25.1 |
| Frank | 59 | 20.8 |
| JACK | 54 | 19.1 |
| QEH | 49 | 17.3 |
| PRUH | 34 | 12.0 |
| UHL | 16 | 5.7 |

| **Table 5.** **Linear regression between pH and the six dependent variables of cardiac power index (W/m2) adjusted by Propensity Score Matching in the six ICUs** | | | |
| --- | --- | --- | --- |
|  | **Regression coefficient** | **95% CI Regression coefficient** | **P- value corrected** |
| Frank | 1.24 | 0.39 – 2.09 | 0.005 |
| LIVER | 0.78 | 0.25 – 1.31 | 0.005 |
| JACK | 1.32 | 0.77 – 1.88 | <0.001 |
| PRUH | -0.27 | -1.14 – 1.16 | >0.200 |
| QEH | 0.52 | -0.13 - 1.16 | 0.114 |
| UHL | 0.54 | -0.01 - 1.10 | 0.053 |

|  |
| --- |
| **Figure 1**. Histograms of APACHE II of pH groups ≤ 7.28 and > 7.28 before propensity score matching. |

|  |
| --- |
| **Figure 2**. Histograms of APACHE II of pH groups ≤ 7.28 and > 7.28 after propensity score matching. |
