# Supplementary material for: Systemic acidemia impairs cardiac function in critically Ill patients
Source: eClinicalMedicine. 2021 Jun 29;37:100956. doi: 10.1016/j.eclinm.2021.100956 (PMC8255172; doi:10.1016/j.eclinm.2021.100956)
Supplement: Supplementary file 2 [file mmc2.docx]

**ACID-BASE DISORDERS WORKING GROUP**

**ACID-BASE DISORDERS WORKING GROUP**

Mcmullen D(6), Clarey E(6), Murphy R (5), Rong G (8), Hopkins P(1), Maciejewski S(1), Hawthorne S(1), Gardner D(1), Rowe O(6), Treus E(5), Vosper D(5), Scally N(5), Howard L(5), Barlow N(5), Ciorcon I(5), McCaffrey B(5), Landridge E(5), Cart H(5), Murray E(5), Wilson J(6), Rufus K(6), Martis V(6), Bica N(5), Armstrong L(1), Clarke J(7), Cole E, Bell C(1), Allen H(1), Brown N(1), Feehan A(1), Peters K(1), Johnson N(1), Anderson P(1), Corcoran E(1), Groves K(1), Arellano M(1), Navarro-Vidal A(6), Linan-Bodi B(6), Bennett F(6), Jaing H(6), Blanca K(6), Sunico R(6), Picard-Sanchez BE(6), Burca T(6), Almeida C(5), Fuster-Munoz AM(6), Suarez-Magtanong J(5), Perez MT(1), Lubomirov-Gueorguiev P(6), Willmott E(5), Smith E(5), Ragasa C(5), Adams R(5), Young S(6), Gillard E(5), Mccaughey P(5), Khaliq W(7), Zaki A(7) and Roshdy A(7).

1. Critical Care Department, King´s College Hospital NHS Trust Foundation. London, United Kingdom.
2. Dialysis Department, VHAGLA Healthcare System. Los Angeles, California, USA.
3. Internal Medicine Department, Hospital Príncipe de Asturias. Alcalá de Henares, Madrid, Spain.
4. Critical Care Department. Gemeinschaftsklinikum Mittelrhein gGmbH. Kemperhof und Ev. Stift St. Martin. Germany.
5. Critical Care Department. Queen Elizabeth Hospital. Lewisham and Greenwich NHS Trust. United Kingdom.
6. Critical Care Department. Princess Royal University Hospital. King´s College Hospital NHS Trust Foundation. London, United Kingdom.
7. Critical Care Department. Lewisham University Hospital. Lewisham and Greenwich NHS Trust. United Kingdom.
8. Department of Medicine Statistics Core. David Geffen School of Medicine, University of California Los Angeles. USA.
